# Urethral Diverticulum in a 45-Year-Old Woman: A Case Report and Review of the Literature

**DOI:** 10.7759/cureus.111284

**Published:** 2026-06-22

**Authors:** Meryem El Bardai, Fatima Zahra Benbrahim, Ismail Chaouche, Hajar Ouazzani, Nizar El Bouardi, Y Lamrani, Meryem Boubbou, Mustapha Maaroufi, Baderdine Alami

**Affiliations:** 1 Department of Radiology, Centre Hospitalier Universitaire (CHU) Hassan II, Fes, MAR; 2 Department of Radiology, Sidi Mohamed Ben Abdallah University, Centre Hospitalier Universitaire (CHU) Hassan II, Fes, MAR; 3 Department of Radiology, Mother and Child Hospital, Centre Hospitalier Universitaire (CHU) Hassan II, Fes, MAR

**Keywords:** atypical chronic urinary symptoms, female, lower urinary tract symptoms, mri, radiology, urethral diverticulum

## Abstract

Female urethral diverticulum involves the herniation of the urethral mucosa into the anterior vaginal wall. These lesions may communicate with the urethra and cause urinary symptoms. Its diagnosis requires clinical and imaging confirmation. We report a case of a 45-year-old woman who presented with dysuria, pollakiuria, post-micturition dribbling, dyspareunia, and recurrent urinary tract infections. Pelvic examination revealed a tender, compressible antrior pelvic mass, expressing purulent discharge upon compression. Pelvic MRI confirmed a diverticular sac communicating with the urethra. Surgical excision achieved complete removal of the diverticulum, followed by multilayer urethral closure. At follow-up, she showed complete symptom resolution. This case of urethral diverticulum with irritative urinary symptoms and dyspareunia demonstrates the diagnostic value of pelvic MRI.

## Introduction

Female urethral diverticulum, also referred to as a periurethral or suburethral pouch, is an outpouching of the urethral mucosa through the muscular wall of the urethra that communicates with the urethral lumen [1]. It is a relatively rare condition in adult women, with an estimated prevalence ranging from 0.6% to 6% [2]. Clinical presentation is often variable and non-specific, which may lead to delayed diagnosis or misdiagnosis. Common symptoms include dysuria, dyspareunia, post-void dribbling, urinary incontinence, and recurrent urinary tract infections [2-5].

Pelvic magnetic resonance imaging (MRI) plays a central role in the evaluation of a suspected urethral diverticulum. It is considered the imaging modality of choice due to its excellent soft-tissue contrast and ability to provide accurate anatomical delineation of the lesion and its communication with the urethra [3,4]. Transvaginal ultrasound may also be used as an initial, widely available, and low-cost imaging modality, although its performance is operator-dependent [6].

The management of a urethral diverticulum depends on symptom severity, size, and anatomical complexity. Options range from conservative follow-up in selected asymptomatic cases to surgical diverticulectomy, which remains the standard treatment in symptomatic patients [2,7]. We report a case of a woman with a urethral diverticulum presenting with chronic urinary symptoms.

## Case presentation

A 45-year-old woman with no significant past surgical history presented with a several-month history of progressive lower urinary tract symptoms. She reported recurrent episodes of dysuria and recurrent urinary tract infections with repeated relapses. These symptoms were associated with persistent perineal pain, a sensation of vaginal heaviness and pressure, and difficulty with complete bladder emptying, significantly affecting her quality of life. She also described intermittent purulent discharge from the urethral meatus, particularly after micturition. Associated symptoms included dyspareunia and intermittent cloudy urine. She denied gross hematuria or fever.

On physical examination, a soft, tender, mobile mass was palpated distinctly on the anterior vaginal wall. Application of digital pressure to this swelling elicited purulent fluid from the external urethral meatus. Initial urinalysis revealed significant pyuria and bacteriuria, consistent with an active infection.

Given the atypical clinical presentation, a pelvic MRI was performed to further diagnose and characterize the underlying anatomy. The MRI revealed a slightly irregular, oval-shaped, multiloculated cystic lesion with a thick wall arising from the anterior wall of the urethra. It communicated with the urethral lumen via a narrow neck. The lesion showed a high signal intensity on T2-weighted sequences with a fluid-fluid level, suggestive of content stasis with possible superinfection (Figure 1). The MRI further excluded urethral calculi, urethrovaginal fistula, and imaging features suggestive of malignant transformation.

**Figure 1:**
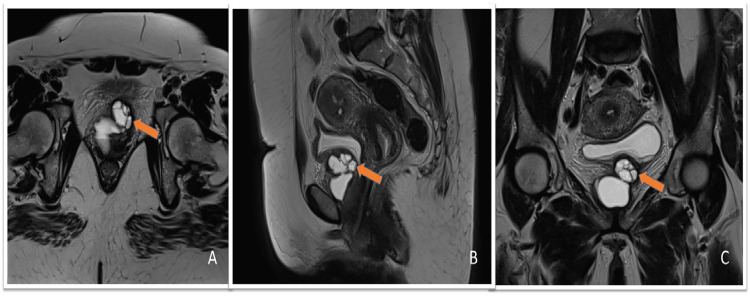
T2-weighted MRI (axial (A), sagittal (B), coronal (C)) demonstrating a multiloculated periurethral diverticulum communicating with the urethral lumen via a narrow neck and containing a fluid–fluid level. The arrow indicates the urethral diverticulum.

Based on clinical and imaging findings, the main differential diagnoses included Skene’s gland cyst, Gartner duct cyst, and periurethral abscess.

The patient subsequently underwent surgical management via a suprapubic approach due to the atypical multiloculated configuration and extensive periurethral involvement of the diverticulum, which made a standard transvaginal approach technically challenging and potentially associated with incomplete excision. Intraoperatively, a multiloculated urethral diverticulum with infected content was identified and completely excised. Careful dissection allowed complete separation of the diverticular sac from the surrounding periurethral tissues, with preservation of urethral integrity. Complete excision of the diverticular neck was achieved, followed by multilayer closure of the urethral defect.

The postoperative course was uneventful. The patient was discharged in good condition and showed progressive resolution of urinary symptoms. At follow-up, she reported significant improvement in dysuria, urinary frequency, and perineal discomfort, with no urinary incontinence, no recurrent infection, and no clinical evidence of recurrence.

Histopathological examination was not available in this case. The diagnosis was confirmed based on clinical presentation, MRI findings, and intraoperative appearance. The chronological sequence of the patient’s clinical presentation, diagnostic workup, and therapeutic management is summarized in Table 1.

**Table 1:** Timeline of clinical presentation, diagnostic workup, and therapeutic management in the present case

Time point	Clinical event
Initial presentation	Chronic lower urinary tract symptoms, including recurrent urinary tract infections, perineal pain, and voiding dysfunction
Physical examination	Palpable anterior vaginal wall mass with expression of purulent discharge from the urethral meatus upon pressure
Laboratory findings	Pyuria and bacteriuria consistent with a urinary tract infection
Imaging	Pelvic MRI demonstrating a multiloculated periurethral cystic lesion communicating with the urethral lumen
Treatment initiation	Empirical antibiotic therapy
Surgical management	Suprapubic diverticulectomy performed without intraoperative complications
Postoperative course	Uneventful recovery
Follow-up	Symptom resolution with no evidence of recurrence

## Discussion

Female urethral diverticulum is a rare but probably underdiagnosed pathology, not least because of the great variability in its clinical presentation [2,5]. Its true prevalence is difficult to establish, ranging from 0.6% to 6% in adult women [2]. Because periurethral masses in women have a broad differential diagnosis, urethral diverticulum should be considered during evaluation [6].

The urethral diverticulum is defined as a herniation of the urethral mucosa through the musculature of the urethra, forming a cavity in communication with the urethral lumen [1]. It is most often located on the posterolateral aspect of the distal or middle urethra, in an intrapelvic position, sometimes palpable anterior to the vagina [1,8]. The most commonly accepted pathophysiological hypothesis is chronic or recurrent infection of the paraurethral glands (Skene's glands), leading to obstruction of their excretory canal, accumulation of secretions, and subsequent suppuration; this suppuration eventually creates a fistula between the infected gland and the urethra, forming a diverticulum [2,8]. Other, less frequent causes include obstetric trauma or iatrogenic events (urethral catheterization, pelvic surgery) [1,9]. The symptomatology of urethral diverticulum is classically described as a triad: dysuria, dyspareunia, and post-micturition discharge [1]. However, this triad is only found in a minority of cases [2,5]. The symptomatology is often broader, including frequency, urinary incontinence, and recurrent urinary tract infections [2,5].

The description/classification of female urethral diverticula is based on anatomo-radiological criteria useful for guiding surgical management, including location (distal, mid, or proximal), number (single or multiple), configuration (saccular, fusiform, or circumferential), and the number and position of ostia communicating with the urethra [3,4,10]. Pelvic MRI is key for this assessment, providing accurate mapping of the diverticulum and its relationship to adjacent structures, which is essential for surgical planning [3,4].

Urethral diverticula are mainly associated with infectious and obstructive complications, such as recurrent urinary tract infections favored by urinary stasis, as well as obstructive symptoms (e.g., urethral stricture or bladder outlet obstruction) varying according to the lesion size [2,5,6]. Although rare, malignant transformation (most commonly adenocarcinoma) may occur in chronic cases [2,9]. Finally, surgical management by diverticulectomy is associated with notable postoperative complications, including urethrovaginal fistula, stress urinary incontinence, and diverticulum recurrence, often related to incomplete excision or inadequate identification of the diverticular neck [2,7].

Management depends on the size, location, symptoms, and complexity of the diverticulum [2,10]. Transvaginal diverticulectomy is the standard technique, involving complete excision of the diverticulum followed by multilayer closure (urethral mucosa, periurethral fascia/muscle, and vaginal wall) [7,10]. In large, complex (multiloculated or circumferential), or recurrent diverticula, interposition of a flap (such as the Martius flap) between the urethra and vagina may reduce the risk of fistula [7]. Other options, though less common, include marsupialization in selected cases or minimally invasive/endoscopic techniques, which remain limited/experimental [2]. Surgical diverticulectomy is associated with high success rates, with reported symptom improvement in approximately 80%-90% of cases [2,7]. Non-surgical management based on surveillance may be considered in rare cases of asymptomatic, small, uncomplicated diverticula [2,10]. MRI is essential before any therapeutic decision is taken because recurrences are often related to incomplete excision or failure to identify the diverticular neck; careful preoperative imaging helps reduce these risks [2,3,4,10]. Some studies describe newer minimally invasive approaches (e.g., endoscopic marsupialization or laser techniques), but these remain experimental and reserved for selected cases [2].

Table 2 summarizes representative published case series and selected case reports of female urethral diverticulum, highlighting clinical presentation, MRI characteristics, and surgical management [3,5,11-17].

**Table 2:** A literature review of female urethral diverticulum studies showing clinical presentation, imaging findings, and management

Study	Study type	Imaging findings	Management
Romanzi et al. (2000) [5]	Case series	Variable periurethral diverticula with nonspecific presentation and frequent diagnostic delay	Transvaginal diverticulectomy
Han et al. (2007) [13]	Case series	MRI demonstrating simple, U-shaped, and circumferential diverticula with defined urethral communication	Transvaginal repair with layered closure
Singla et al. (2013) [3]	Imaging review	MRI characterizing diverticular morphology and urethral communication	Imaging-based diagnosis; surgical management planned accordingly
Porten & Kielb (2008) [15]	Review article	Imaging (including MRI) defining the extent, configuration, and urethral neck of diverticula	Standard transvaginal diverticulectomy described
Pirpiris et al. (2022) [14]	Multicenter case series	MRI is the standard modality for diagnosis and preoperative planning, defining complexity and extent	Transvaginal diverticulectomy, with Martius flap interposition in selected cases
Rachid et al. (2025) [11]	Case report	MRI-confirmed urethral diverticulum with clear communication with the urethral lumen	Transvaginal diverticulectomy
Noor Affendi et al. (2025) [12]	Case report	Diverticulum complicated by intradiverticular calculus on imaging	Diverticulectomy with stone extraction
Vakili et al. (2003) [17]	Case report	Cystic periurethral lesion communicating with the urethra on imaging	Transvaginal diverticulectomy
Xie et al. (2020) [16]	Case report	Large cystic periurethral diverticulum identified on imaging	Transvaginal diverticulectomy
Present case (2026)	Case report	Multiloculated infected urethral diverticulum with fluid–fluid level and complex MRI anatomy	Suprapubic diverticulectomy

Recent literature, including case reports published in 2025, has expanded the clinical spectrum of female urethral diverticulum by describing complicated presentations, including diverticula associated with intradiverticular calculi and recurrent urinary tract symptoms. These reports highlight that inflammatory or obstructive complications may occur in selected cases and reinforce the heterogeneous nature of this condition. However, they do not typically describe severely infected or multiloculated diverticula as seen in the present case [11,12]. Overall, the literature indicates that most urethral diverticula are simple or U-shaped and are managed successfully with a standard transvaginal approach [5,13,14]. More complex morphological variants remain less frequently reported and may require individualized surgical strategies. In contrast, the present case demonstrates an exceptionally complex and atypical multiloculated infected urethral diverticulum with a fluid-fluid level and marked periurethral distortion on MRI, a configuration rarely reported in the literature. Due to the complexity of the lesion and associated infection, a suprapubic surgical approach was required instead of the standard transvaginal route.

Taken together, these findings emphasize that although MRI is essential for diagnosis and surgical planning, female urethral diverticulum remains a highly heterogeneous entity in which optimal management must be individualized according to anatomical complexity and intraoperative findings rather than standardized surgical algorithms.

This study is not without limitations. Histopathological confirmation was not available; however, the diagnosis was supported by concordant clinical findings, characteristic MRI features, and intraoperative observations. In addition, as a single case report, the findings are inherently limited in terms of generalizability. Furthermore, long-term imaging follow-up was not performed, precluding definitive assessment of recurrence. Despite these limitations, this case highlights an unusual presentation of urethral diverticulum and emphasizes the importance of individualized diagnostic evaluation and tailored surgical management.

## Conclusions

Female urethral diverticulum is an uncommon and often underdiagnosed cause of chronic lower urinary tract symptoms in women. Its clinical presentation is highly variable, which may contribute to delayed diagnosis. This case highlights the importance of considering this diagnosis in patients with recurrent urinary tract infections and nonspecific urinary symptoms. MRI may provide valuable anatomical information for preoperative assessment, although imaging findings should always be interpreted in conjunction with clinical and physical examination. Surgical management is generally considered the standard treatment for symptomatic urethral diverticulum, but the surgical approach should be individualized, particularly in complex or atypical cases. Overall, this case underscores the importance of a multidisciplinary and stepwise approach to diagnosis and management.
